# Effect of chest wall loading during supine and prone position in a critically ill covid-19 patient: a new strategy for ARDS?

**DOI:** 10.1186/s13054-021-03865-2

**Published:** 2021-12-20

**Authors:** Sergio Lassola, Sara Miori, Andrea Sanna, Rocco Pace, Sandra Magnoni, Luigi Vetrugno, Michele Umbrello

**Affiliations:** 1 SC Anestesia E Rianimazione 1, Ospedale Santa Chiara, Trento, Italy; 2 Department of Anesthesiology, Critical Care Medicine and Emergency, SS, Annunziata Hospital, Chieti, Italy; 3 Department of Medical, Oral and Biotechnological Sciences, University of Chieti-Pescara, Chieti, Italy; 4 SC Anestesia E Rianimazione II, Ospedale San Carlo Borromeo, ASST Santi Paolo e Carlo, Milan, Italy

**Keywords:** COVID-19 (C- ARDS), Esophageal pressure, External chest wall compression, Lung protection, Mechanical ventilation, Prone position

Dear Editor,

We read with great interest the review by Gattinoni and Marini [1]. In their paper, the authors postulate a positive effect on respiratory mechanics of local chest wall compression over the sternum or the abdomen of patients with severe ARDS, which is supposed to improve the tidal lung compliance and transpulmonary pressure.

The global pandemic of SARS-CoV-2 infection, and the consequent coronavirus disease 2019 (COVID-19), the most concerning complication, of which is acute hypoxemic respiratory failure, led to a surge in patients requiring mechanical ventilation and ICU admission [2]. In a small but significant part of such patients, conventional lung protective ventilation is not sufficient to relieve hypoxemia, and other strategies should be taken into account.

Prone positioning is an established strategy to improve oxygenation in severe ARDS, and its application was associated with a reduction in mortality rate [3]. Placing patients into prone position induces a more uniform distribution of tidal volume by reversing the vertical pleural pressure gradient. In addition, prone position decreases the superimposed pressure of both the heart and the abdomen on the dorso-caudal regions of the lungs [4]. On the contrary, pulmonary perfusion remains preferentially distributed to the dorsal lung regions, thus improving overall alveolar ventilation/perfusion matching. Moreover, the larger lung tissue mass suspended from a wider dorsal chest wall effects a more homogeneous distribution of pleural pressures throughout the lung, which in turn reduces abnormal strain and stress development. This is believed to avoid the development of ventilator-induced lung injury and may partly explain the reduction in mortality in severe ARDS [5].

Some case reports have sparked curiosity about using additional weights on the chest wall to improve lung compliance and thus ameliorate hypoxemia and respiratory mechanics [6, 7]. We report the effect of loading and unloading the chest wall during prone and supine position in a critically ill patient with COVID-19-related ARDS (C-ARDS).

A 65-year-old patient with class 2 obesity and no relevant comorbidities needed intubation and mechanical ventilation due to C-ARDS. No previous lung disease was reported in his medical history. His respiratory mechanics progressively worsened despite protective ventilation (5 mL/Kg PBW). PEEP was 12 cmH_2_O, respiratory rate 18/min, FiO_2_ 0.7. Respiratory system elastance was > 50 cmH_2_O/L, and airway driving pressure was 22 cmH_2_O. It was necessary to institute ultra-protective lung ventilation (3.5 mL/Kg PBW) with extracorporeal carbon dioxide removal (ProLUNG®, ESTOR, Pero, Milano, Italy) at a blood flow of 400 ml/min and a fresh gas flow of 15 l/min oxygen. An esophageal balloon catheter (NutriVent®, SEDA, Mirandola, Modena, Italy) was positioned to investigate partitioned respiratory mechanics, and a pulmonary artery catheter was inserted.

Compression of the chest wall (over both sternum and ribs) with a sand bag was performed in the supine position, then the patient was placed in the prone position, and the sand bag was applied again. Table 1 shows the respiratory mechanics, gas exchange and hemodynamic parameters in the different conditions; Fig. 1 shows the lung elastance, alveolar dead space and oxygenation in the different conditions.

**Table 1:** Lung mechanics, ventilation and hemodynamic parameters during supine and prone position while loading and unloading the chest wall

Parameters	Supine		Prone	
	Weight off	Weight on	Weight off	Weight on
End-inspiratory airway pressure (cmH_2_O)	28	24	25	20
End-inspiratory transpulmonary pressure (cmH_2_O)	26	20	23	13
Airway driving pressure (cmH_2_O)	16	12	13	8
Respiratory system elastance (cmH_2_O/L)		41	45	28
Chest wall elastance (cmH_2_O/L)	3	7	3	10
Lung elastance (cmH_2_O/L)	52	34	42	18
Venous admixture (%)	37	34	34	29
PaO_2_/FiO_2_	104	122	115	160
pH	7.29	7.31	7.31	7.34
SvO_2_ (%)	79	78	77	76
PvO_2_ (mmHg)	47	46	44	44
PaCO_2_ (mmHg)	59	56	54	52
EtCO_2_ (mmHg)	36	37	38	39
Alveolar dead space (%)	39	34	29	25
VCO_2_ Membrane lung (ml/min)	139	133	134	112
VCO_2_ Natural lung (ml/min)	97	106	105	130
VO_2_ (ml/min)	268	272	277	283
Heart rate (bpm)	83	84	86	87
Arterial blood pressure (mmHg)	118/62	114/64	115/65	118/68
Pulmonary wedge pressure (mmHg)	8	10	10	14
Pulmonary arterial pressure (mmHg)	39/13	37/14	34/15	38/18
Cardiac Index (l/min/m^2^)	3.5	3.5	3.3	3.2

VCO_2_, carbon dioxide production; VO_2_, oxygen consumption

**Fig. 1:**
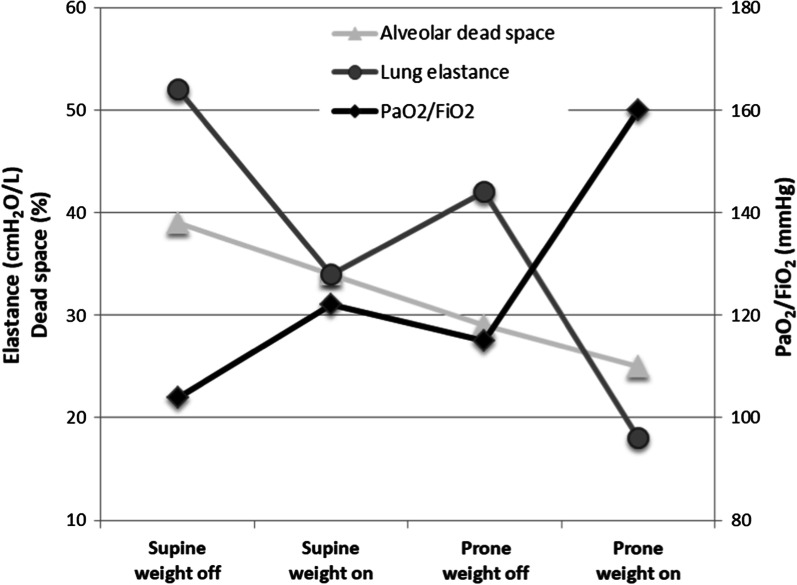
Lung mechanics, alveolar dead space and oxygenation during supine and prone position while loading and unloading the chest wall

In the supine position, external chest wall compression increased the chest wall elastance and reduced the lung elastance, ​​with a consequent reduction in the end-inspiratory transpulmonary pressure and therefore in the stress applied to the lung. Moreover, despite an unmodified minute ventilation, PaCO_2_ decreased, as did the alveolar dead space. Interestingly, a reversal of CO_2_ elimination percentages between natural and membrane lung was found. Third, venous admixture decreased, and oxygenation increased. In summary, chest wall loading likely led to a reduction in hyperinflation in the non-dependent lung region. Redistribution of ventilation and pulmonary blood flow is likely to account for some of the improved gas exchange during chest wall loading. Notably, the physiologic effects of external chest wall compression in the supine position were very similar to those of prone positioning. Our findings are similar to those reported by Carteaux et al. [8, 9]. Interestingly, application of chest wall loading in the prone position led to a further improvement of lung mechanics and oxygenation, confirming the recent finding of an improved compliance and lower plateau and driving pressure after sustained compressive force applied to the dorsum of the passive and prone patients with severe cARDS during controlled mechanical ventilation, which suggests end-tidal overinflation within the aerated part of the diseased lung despite the already compressed anterior chest wall of prone positioning [10].

It is possible that in the late phase of C-ARDS [11], the application of a weight (sand bag) on the chest in both the supine and prone position improves respiratory mechanics by reducing airway and transpulmonary driving pressures [8, 9, 12]. This maneuver is likely associated with a decrease in non-dependent lung region overdistension and an increase in dependent region recruitment of aerated lung units, leading to a more homogeneous tidal ventilation [8], adding a further element that improves lung protective ventilatory strategies [1].

Our case confirms the previous findings of chest wall loading in the supine position and adds evidence also to patients in the prone position. However, the possible role of chest loading is not generalizable to all ARDS patients, as some may not respond to this maneuver, and these initial observations require further investigation, even in little-explored areas such as the role of abdominal binding in responsive patients [10].

According to Gattinoni and Marini, we suggest that chest loading maneuver should be tested in all patients suffering from ARDS, applying it only in responders. Large further studies are needed to verify if this approach shares with prone positioning the same positive effect on patient outcome.

## Data Availability

All data generated or analyzed during this study are included in this published article.
